# Re-alignment of the unmapped reads with base quality score

**DOI:** 10.1186/1471-2105-16-S5-S8

**Published:** 2015-03-18

**Authors:** Xiaoqing Peng, Jianxin Wang, Zhen Zhang, Qianghua Xiao, Min Li, Yi Pan

**Affiliations:** 1 School of Information Science and Engineering, Central South University, 410083 Changsha, China; 2 Department of Computer Science, Georgia State University, 30302-4110 Atlanta, USA

**Keywords:** re-alignment, unmapped reads, base quality score

## Abstract

**Motivation:**

Based on the next generation genome sequencing technologies, a variety of biological applications are developed, while alignment is the first step once the sequencing reads are obtained. In recent years, many software tools have been developed to efficiently and accurately align short reads to the reference genome. However, there are still many reads that can't be mapped to the reference genome, due to the exceeding of allowable mismatches. Moreover, besides the unmapped reads, the reads with low mapping qualities are also excluded from the downstream analysis, such as variance calling. If we can take advantages of the confident segments of these reads, not only can the alignment rates be improved, but also more information will be provided for the downstream analysis.

**Results:**

This paper proposes a method, called RAUR (Re-align the Unmapped Reads), to re-align the reads that can not be mapped by alignment tools. Firstly, it takes advantages of the base quality scores (reported by the sequencer) to figure out the most confident and informative segments of the unmapped reads by controlling the number of possible mismatches in the alignment. Then, combined with an alignment tool, RAUR re-align these segments of the reads. We run RAUR on both simulated data and real data with different read lengths. The results show that many reads which fail to be aligned by the most popular alignment tools (BWA and Bowtie2) can be correctly re-aligned by RAUR, with a similar *Precision*. Even compared with the BWA-MEM and the *local *mode of Bowtie2, which perform local alignment for long reads to improve the alignment rate, RAUR also shows advantages on the *Alignment rate *and *Precision *in some cases. Therefore, the trimming strategy used in RAUR is useful to improve the *Alignment rate *of alignment tools for the next-generation genome sequencing.

**Availability:**

All source code are available at http://netlab.csu.edu.cn/bioinformatics/RAUR.html.

## Introduction

Next-generation genome sequencing (NGS) technologies, including Illumina/Solexa and AB/SOLiD, generate billions of short reads (25-200 bp) and become more and more popular. Based on NGS technologies, a variety of biological applications are developed. In many large projects, resequencing and read mapping are extensively used, such as 1000 Genome Project[1] and ENCODE [2]. Recently various high-throughput approaches based on bisulfite conversion combined with NGS have been developed and applied for the genome wide analysis of DNA methylation [3]. Resequencing [4], disease genome study [5], and identification of genetic variants [6,7] are also benefited greatly by NGS. For most applications and analysis, assembly and alignment are the first step once sequencing reads are obtained. When reference genomes are not available, assembly will be used to construct genomes and many algorithms have been proposed, such as [8]. The alignment algorithms are applied when reference genomes are available. However, there are many challenges to accurately map the reads to the genome, due to the sequencing errors with an overall per base error rate around 1-2% [9], repeats in the reference genome and differences between the donor and reference genomes.

In recent years, many short read alignment algorithms have been developed to address these challenges, different in speed, memory, accuracy, and alignment strategy [10,11]. There are two main strategies adopted in them. One strategy is spaced seeds, and the representative alignment algorithms are known as MAQ [12] and SOAP [13]. The other one is Burrow-Wheeler Transform [14], and the representative alignment algorithms are BWA [15], Bowtie2 [16], and SOAP2 [17]. Although these alignment algorithms are more and more efficient and accurate, there are a portion of reads which are not mapped at all by the alignment tool or the mapping quality scores are less than the threshold.

### The mapping quality and the related works

Mapping quality was firstly proposed in MAQ [12], which is an indicator of the likelihood that a mapping is accurate. Later on, many alignment tools also report mapping qualities for their alignments. The calculation of mapping quality is related to "uniquenes". An alignment is unique if it has a much higher alignment score than all the other possible alignments. In another word, the bigger the gap between the best alignment's score and the second-best alignment's score, the more unique the best alignment, and the higher its mapping quality should be.

Mapping quality is important to the downstream analysis, like variance calling. For instance, a variant caller might choose to ignore evidence from alignments with mapping quality less than 10. However, in almost all the state-of-the-art alignment tools, the mapping quality scores do not correlate well with the actual likelihood that a mapping is accurate[11]. Many accurate mappings are generally reported with quality 0, and many inaccurate mappings are reported with high-quality scores. The RMAP algorithm [18] is proposed to improve mapping accuracy by incorporating base-call quality scores to weight mismatches. Furthermore, Ruffalo *et al*. [19] use a machine learning approach to re-calculate the mapping qualities of the short read mappings which are more accurate than those reported by the available alignment tools.

### The coming of unmapped reads

The re-calculation of mapping quality of the mappings can make the mapping quality more reliable and promote the accuracy to some extent. However, it can do nothing for the reads which are reported as unmapped.

For most alignment tools, the edit distances or the allowed mismatches are limited, thus some reads can not be mapped if the number of mismatches in any hit exceeds the allowable differences. Given a read of length *m*, BWA [15] only tolerates at most *k *differences (mismatches or gaps) in a hit, where *k *is chosen such that < 4% of *m*-long reads with 2% uniform base error rate. With this configuration, for 15-37 bp reads, *k *equals 2; for 38-63 bp, *k *= 3; for 64-92 bp, *k *= 4; for 93-123 bp, *k *= 5; and for 124-156 bp reads, *k *= 6. That is to say, the reads with differences more than *k *in any hits will be unmapped.

Some trimmed-like strategies appear in some alignment programs and try to handle the problem. For example, in local read alignment mode, Bowtie2 [16] might "trim" or "clip" some read characters from one or both ends of the alignment to maximize the alignment score. The local read alignment can improve the *Alignment rate *at some extend. However, the false positive sites are also introduced by maximizing the alignment score which will affect the alignment accuracy, since the maximum alignment score can't guarantee that high quality bases are involved. BWA-MEM [20] is a new alignment algorithm, which can perform local alignment and is robust to sequencing errors and applicable to a wide range of sequence lengths.

### Our contribution in this article

The unmapped reads also contain many information which is important to the downstream analysis. Thus in this article, we propose a method named (RAUR) to re-align these unmapped reads. A trimming strategy used in RAUR is to figure out the longest and most confident and informative segment of a read based on base quality score. It adopts an iterative progress to trim the unmapped reads until the reads can be confidently mapped or can't be mapped in the whole progress. RAUR can combine with any alignment tool to improve the alignment rate. In our experiments, RAUR is combined with BWA [15] and Bowtie2 [16] separately, and run on both the simulated data and real data with different read lengths. By comparing the *Precision *and *Alignment rate*, we can find out that RAUR can improve the *Alignment rate *of each alignment tool greatly, while the *Pecision *are still comparative with those of the original alignment tool. Furthermore, in some cases, it has comparative or better performance than BWA-MEM and the local read alignment mode of Bowtie2, which also adopt trimmed-like strategies.

## Methods

In this section, we investigate the correlation between the low base quality scores and sequencing errors. Based on the investigation, the trimming strategy adopted in RAUR is presented in details. Then, RAUR algorithm is described.

### Base quality scores distribution

Quality score measures the probability that a base is called incorrectly. With sequencing by synthesis technology, each base in a read is assigned a quality score by a phred-like algorithm [21], similar to that originally developed for Sanger sequencing experiments. The quality score of a given base, *Q*, is defined by Equation 1.

(1) $$Q=-10{\text{log}}_{10}\left(e\right)$$

where *e *is the estimated probability of the base call being wrong. Thus, a higher quality score indicates a smaller probability of error. A quality score of 10 represents an error rate of 1 in 10, with a corresponding call accuracy of 90%; a quality score of 20 represents an error rate of 1 in 100, with a corresponding call accuracy of 99%; a quality score of 30 represents an error rate of 1 in 1000, with a corresponding call accuracy of 99.9%. In this paper, a base quality score ≥ 20 is considered as a high base quality, otherwise it is a low base quality.

**Figure 1:**
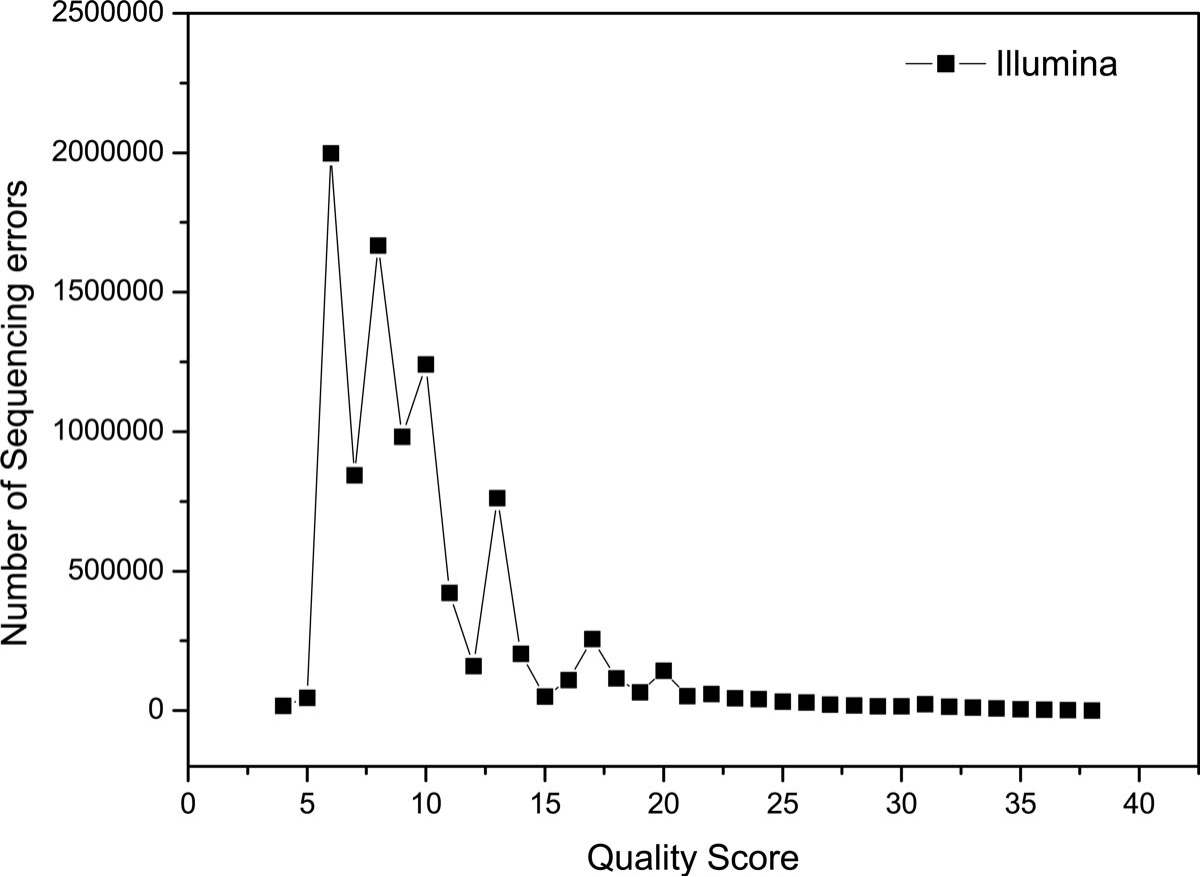
**The quality score distribution of sequencing errors**. The 10 million reads of Illumina's Solexa with length 50-bp simulated by ART, and each base in a read is assigned a quality score by a phred-like algorithm. X represents the quality scores ranging from 0 to 40, and Y represents the number of sequencing error corresponding for each X value.

**Figure 2:**
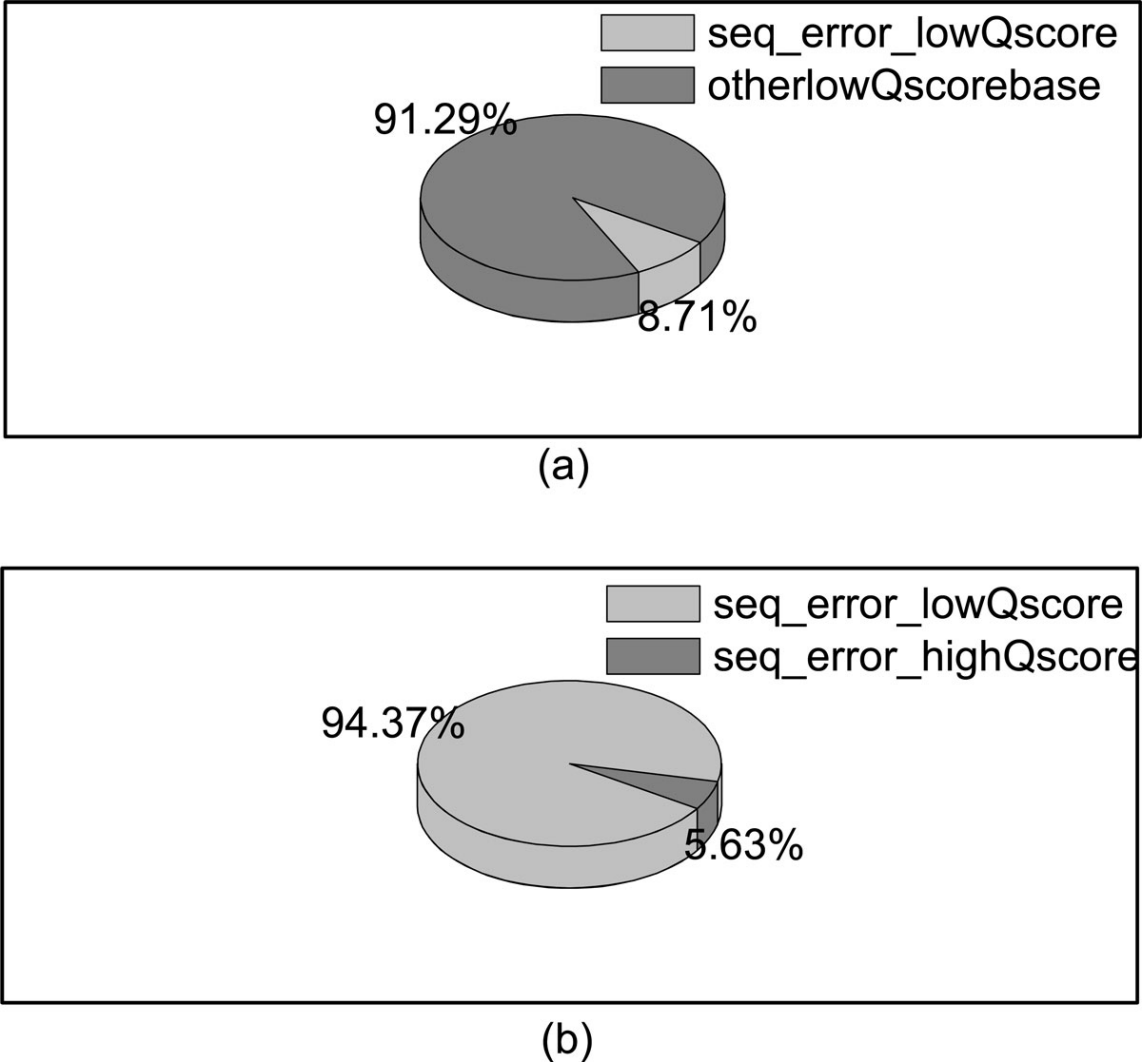
**Percentage of sequencing errors in bases with Quality score below 20**. (a) shows the percentage of sequencing errors with quality scores lower than 20 in all the bases with quality scores lower than 20; (b) shows the percentage of sequencing errors with quality scores lower than 20 in all the sequencing errors.

Sequencing errors are one of the main resources for mismatches. The differences between the individual genome and the reference genome are the other resource for mismatches or gaps in alignment. We investigate the quality score of sequencing errors of ILLUMINA sequencing reads with length 50-bp simulated by ART [22]. As shown in Figure 1, we can observe that, the base quality scores of the majority of sequencing errors are lower than 20. On the other side, majority of the bases (above 90%) with low base quality scores (≤ 20) are not sequencing errors, as shown in Figure 2.

### The strategy of trimming

There is a saying that the more things you do, the higher possibility you will make a mistake. Similarly, more bases considered, more sequencing errors will be encountered, which may ruin the alignment. With the number of mismatches or the edit distance greater than the allowed value, some reads will be unmapped by the alignment tools, or are mapped with low mapping qualities. These reads are excluded from downstream analysis. However, some confident segments of these reads can be used in variance calling. The first and most important step to make use of the unmapped reads is to figure out the most confident and informative segment of an unmapped read, which can be aligned correctly. This step is called trimming.

The purpose of trimming is to control the number of possible mismatches in the alignment. Mismatches in alignment can be sequencing errors and variances. Given a segment with *K *low quality bases, the maximum number of possible mismatches is *K*+*b*, and the minimum number is 0, where *b *is the number of possible variances. From Figure 2, we can know that the probability that all the *K *low quality bases in the segment are sequencing errors is small. Furthermore, Sachidanandam *et al*. [23] found out that it is nearly in 1 kb that there is a SNP, which indicates in a short read, *b *is ≤ 1. Thus, an alignment tool which allows *K *edit distances in a read, can align a segment with *K *low quality bases confidently. Additionally, to align uniquely, the length of the segment should be long enough. Thus, our aim of trimming is to find the longest segment with no more than *K *low quality bases, which can be aligned uniquely.

The details of trimming is illustrated as Algorithm 1. The inputs are unmapped reads, and parameter *K*. *K *is the number of low quality bases allowed in the segment. For each read, the positions of the bases with low qualities in the read are stored in a array. A segment of a read is several successive bases. Then we check the lengths of segments in the read containing *K *low quality bases. Each unmapped read is undertook the trimming in RAUR, and can be represented by a longest segment(or called a trimmed read) under the parameter *K*. The longest segments will be output in the same format as the original unmapped reads. The start position and the end position of a trimmed read in the original unmapped read are recorded, which can be used to deduce the position of an original unmapped read by using these information.

**Algorithm 1 **Trimming

1: **Input: **reads in fastq format, parameter *K*;

2: **Output: **trimmed reads in fastq format;

3:

4: **Process:**

5: **for **each read *R ***do**

6:                     ▹ find the positions of low quality score

7:   *N_Low *= 0,*i *= 0,*Low_position *= [];

8:   *Max_length *= 0,*Max_start *= 0,*Max_end *= 0;

9:   **for **each base *i *∈ *R ***do**

10:      **if ***i *has a low base quality **then**

11:         *Low_position*[*N_Low*++] = *i*

12:      **end if**

13:

14:   **end for**

15:   **if ***N_Low *≤ *K ***then**

16:      output *R *in fastq format

17:      next

18:   **end if**

19:            ▹find the longest segment with *K *low quality bases

20:   **for ***S *= 0;*S *≤ *N Low *− *K*;*S*++ **do**

21:      *length *= 0,*start *= 0,*end *= 0,*j *= *S *+ *K*;

22:      **if ***S *≥ 1 **then**

23:         *start *= *Low_position*[*S*-1]+1;

24:      **else**

25:         *start *= 0;

26:      **end if**

27:      **if ***j *<*N Low ***then**

28:         *end *= *Low_position*[*j*]-1;

29:      **else**

30:         *end *= *R.length*-1;

31:      **end if**

32:      *length *= *end*-*start*+1

33:      **if ***length *>*Max_length ***then**

34:         *Max_start *= *start*

35:         *Max_end *= *end*

36:         *Max_length *= *length*

37:      **end if**

38:   **end for**

39:   Output substr(*R*,*Max_start*,*Max_end*,*Max_length*) in fastq format

40: **end for**

### RAUR algorithm

The process of RAUR is illustrated in Algorithm 2. Firstly, reads are aligned by an alignment program. Then the unmapped reads and the unconfident mapped reads (with mapping quality less than 10) [15] are the input of the loop. RAUR makes every effort to find out the longest and mappable segments of these reads by decreasing the values of *K *of the loop. The parameter *K *is used to control the number of low quality bases allowed in the trimmed reads. In all experiments of this paper, *K *is set as 8. For each iteration, the first step is to trim each unmapped reads into a longest segment (trimmed reads) containing *K *low quality bases. Then align these trimmed reads by the alignment program. When the trimmed reads with *K *low quality bases cannot be aligned or confidently mapped, their original reads are the input of the next loop with *K *= *K*-1. The whole process will stop when *K *= 0. Thus, for each read, it either can be confidently mapped with a certain value of *K *or can't be mapped with any value of *K*.

**Figure 3:**
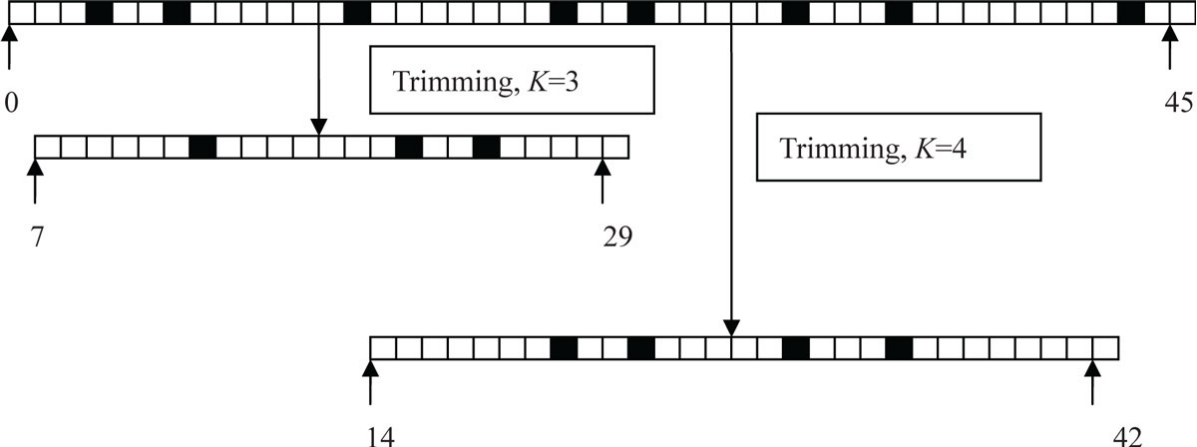
**An example of trimming**. The consecutive squares represent the bases of a read with 45 bp, where the black color squares denote the bases with low quality scores, and in contrast the white color squares are the bases with high quality scores. There are eight bases with low quality scores in the read. When *K *= 4, the read is trimmed into a longest segment which contains four low quality bases, and when *K *= 3, the read is trimmed into a longest segment which contains three low quality bases.

Figure 3 shows an example of trimming a read. The consecutive squares represent the bases of a read with 45 bp, where the black color squares denote the bases with low quality scores, and in contrast the white color squares are the bases with high quality scores. There are eight bases with low quality scores in the read. When *K *= 4, the longest segment of the read starts at position 14 of the original read, and ends at position 42, containing four low quality bases. When the trimmed read can't be aligned, *K *is decreased by 1, and the trimming algorithm search for the longest segment containing three low quality score bases. The start position of the longest segment is 7, and end position is 29. The trimming will stop when the read can be confidently mapped or *K *= 0. In our experiments, the initial value of *K *is set as 8.

**Algorithm 2 **RAUR

1: **Input: **reference sequence, illumina *Reads *in fastq format, parameter *K*(*K *> 0);

2: **Output: ***alignment_file *in sam format;

3:

4: **Process:**

5: Align *Reads *against reference sequence with an aligner;

6:

7: Figure out the unmapped reads and reads with *mapping quality *≥10 and write into file *unmapped_R_eads*

8:

9: **for ***K_low *= *K*;*K_low *> 0;*K_low *= *K_low *- 1 **do**

10:               ▹ Trim reads into longest segments with *K_low *low quality bases

11:      *K_low_Reads *= Trimming(*unmapped_Reads*, *K_low*);

12:

13:      Align *K_low_Reads *against reference sequence with an aligner;

14:

15:      Figure out the unmapped reads and reads with *mapping quality *≥10 and write their original reads into file *unmapped_R_eads*

16:

17: **end for**

## Results

### Evaluated programs and Evaluation metrics

To demonstrate the efficiency of RAUR, two alignment programs are involved in the experiments: BWA(v0.7.5)[15,20], and Bowtie(v2.0.4)[16], which are BWT-based short read alignment tools. RAUR combines each alignment program separately to re-align the unmapped reads and the unconfident mapped reads. RAUR(BWA) and RAUR(Bowtie2) denote the alignment program combined in RAUR. The two alignment programs are run independently as the control group. BWA-MEM algorithm and the local mode of Bowtie2 are sensitive to align longer reads, such as 70 bp-1 Mbp query reads. For further comparison, BWA-MEM [20] (denoted as BWA(mem)) and the local mode of Bowtie2 (denoted as Bowtie2(local)), which perform local alignment for long reads to improve the alignment rate, are run on the datasets with read length greater than 70. For all the alignment programs, the default options are adopted, and the value of *K *in RAUR is initiated as 8.

To evaluate the performance of different alignment programs, the *Alignment rate *and *Precision *are compared. After alignment, all the reads can be classified into three classes, confidently mapped reads, unconfidently mapped reads and the un-mapped reads. The threshold of mapping quality score to differentiate confident mappings and unconfident mappings is set as 10 for all the alignment programs. *Alignment rate *is the fraction of confidently mapped reads to all the reads defined as Equation 2. On simulated data, we can know the correct chromosomal coordinates of the alignment and the *Precision *can be measured. According to the correct chromosomal coordinates, confidently mapped reads can be classified into confidently and correctly mapped reads and confidently but incorrectly mapped reads. Thus, *Precision *is defined as the fraction of confidently and correctly mapped reads among all the confidently mapped reads, calculated according to Equation 3.

(2) $$Alignment\text{\_}rate=\frac{CN}{N}$$

(3) $$Precision=\frac{CCN}{CN}$$

where *N *is the number of total reads, *CN *is the number of confidently mapped reads with mapping quality ≥ 10, and *CCN *is the number of confidently and correctly mapped reads.

### Simulated data and performance

On simulated data, we can know the correct chromosomal coordinates of the alignment and the evaluation is straightforward. We simulate reads from the whole human genome using ART [22], which simulates sequencing reads by mimicking real sequencing process with empirical error models or quality profiles summarized from large recalibrated sequencing data. In this paper, ART is used to simulates sequencing reads of Illumina's Solexa. Six datasets, including both single-end reads and paired-end reads, are generated by ART against the reference genome of human Hg19, with read length 50-bp, 75-bp and 100-bp, respectively. Each dataset contains more than 1 million reads. And then, the alignment programs map the reads back to the human genome. As the exact coordinate of each read is known, it is able to calculate the *Precision *of the alignments. Table 1 and 2 show the *Alignment rate *and *Precision *of each alignment programs on single-end datasets and paired-end datasets, respectively.

**Table 1:** The *alignment rate *and *precision *of each alignment method on single-end simulated data with different read length.

	50-bp		75-bp		100-bp	
			
	*Align.Rate*(%)	*Prec*(%)	*Align.Rate*(%)	*Prec*(%)	*Align.Rate*(%)	*Prec*(%)
BWA	79.4737	99.7359	73.7762	99.7975	30.3573	99.7208
BWA(mem)	-	-	82.8545	99.8912	83.0971	99.8004
RAUR(BWA)	83.5165	99.3132	86.5413	99.1875	87.8022	99.1834
Bowtie2	74.8779	99.6313	77.8351	99.7501	71.4820	99.8918
Bowtie2(local)	-	-	85.206	95.8658	82.3958	95.5368
RAUR(Bowtie2)	83.0495	98.2984	85.3716	98.3442	86.8258	98.2009

There are 7,740,912 simulated single-end reads with length 50-bp, 5,156,962 with length 75-bp, and 3,868,843 with length 100-bp.

**Table 2:** The *alignment rate *and *precision *of each alignment method on paired-end simulated data with different read length.

	50-bp		75-bp		100-bp	
			
	*Align.Rate*(%)	*Prec*(%)	*Align.Rate*(%)	*Prec*(%)	*Align.Rate*(%)	*Prec*(%)
BWA	89.0737	99.6436	91.6370	99.8411	34.7815	99.6929
BWA(mem)	-	-	97.3837	99.8505	96.6372	99.6355
RAUR(BWA)	94.8130	99.2667	96.8181	99.7171	97.0432	98.9618
Bowtie2	84.2039	99.8432	88.0185	99.9409	77.5385	99.9537
Bowtie2(local)	-	-	95.0565	98.0066	90.5642	96.8716
RAUR(Bowtie2)	96.6203	98.2447	96.9858	99.1592	96.8685	98.7567

There are 996,739 pairs simulated paired-end reads with length 50-bp, 1,541,980 pairs with length 75-bp, and 1,156,184 pairs with length 100-bp.

As shown in Table 1 for the simulated single-end reads with length 50 bp, the *Alignment rate *of BWA and Bowtie2 are about 74% and 79%, respectively, while the *Alignment rate *of RAUR(BWA) and RAUR(Bowtie2) are about 83%. It means about 4% and 9% reads can be re-aligned by RAUR. The *Precision *of RAUR(BWA) is comparative with that of BWA and Bowtie2, whose *precision *are above 99%, while the *Precision *of RAUR(Bowtie2) has a little decrease. For the 75-bp reads and 100-bp reads, the *Alignment rate *of RAUR(BWA) and RAUR(Bowtie2) not only outperform BWA and Bowtie2, but also show advantages when compared with BWA(men) and Bowtie2(local). Although in theory BWA works with arbitrarily long reads, its performances are degraded on long reads especially when the sequencing error rate is high. The *Alignment rate *of RAUR(BWA) are about 13% more and 47% more than those of BWA on the 75-bp reads and 100-bp reads, and about 3% more and 4% more than those of BWA(men). The *Precision *of RAUR(BWA) are above 99%, which are comparative with those of BWA and B-WA(men). Compared with Bowtie2, the *Alignment rate *of both RAUR(Bowtie2) and Bowtie2(local) on the 75-bp reads and 100-bp reads are improved, however, their *Precision *decrease to about 98% and 95%, respectively.

The performance of each alignment program on paired-end reads with different read lengths are compared, as shown in Table 2. Compared with the single-end reads with the same read length, the *Alignment rate *of each alignment program on paired-end reads are much higher. The *Alignment rate *of BWA and Bowtie2 are about 84% and 89% on 50-bp paired-end reads, and 91% and 88% on 75-bp paired-end reads, respectively. However, the *Alignment rate *of BWA on 100-bp paired-end reads is as low as that of BWA on 100-bp single-end reads. In contrast, the *Alignment rate *of RAUR(BWA) and RAUR(Bowtie2) are above 94% on paired-end reads with different read lengths. Compared with Bowtie2(local), not only the *Alignment rate *but also the *Precision *of RAUR(BWA) and RAUR(Bowtie2) are greater than those of Bowtie2(local) on both 75-bp paired-end reads and 100-bp paired-end reads. However, the performances of BWA(men) are slightly better than RAUR(BWA) on *Alignment rate *or *Precision*.

From Table 1 and 2, it is easy to find out that with longer read length, the numbers of unmapped reads are increasing, and the *Alignment rate *of BWA and Bowtie2 are declined, while RAUR(BWA) and RAUR(Bowtie2) can dramatically improve the *Alignment rate *by re-aligning the unmapped reads. Furthermore, we can observe that RAUR(BWA) and RAUR(Bowtie2) can achieve higher *Alignment rate *on datasets with longer read length, and the *Precision*s are above 98%. It indicates that for long reads there exist some fragments whose mapping positions can correctly deduce the mapping positions of the original reads, and RAUR can figure out these most informative fragments to be aligned. Table 3 and 4 list the numbers of re-aligned reads which are actually *TP *(true positive), and *FP *(false positive) from single-end simulated datasets and paired-end simulated datasets, respectively. Most of re-aligned reads are eventually *TP*. In Table 1 and 2, the alignment rate of RAUR(Bowtie2) are improved, while the precision of RAUR(Bowtie2) are less than those of RAUR(BWA). The reason lies in the different strategies of Bowtie2 and BWA to perform gapped alignment. BWA pays different penalties for mismatches, gap opens and gap extensions. Bowtie2 combines the full-text minute index-assisted seed alignment and SIMD-accelerated dynamic programming to perform sensitive gapped alignment without incurring serious computational penalties. For Illumina reads, there are only substitution errors seldom indel errors. Since the simulated sequencing reads of Illumina's Solexa are generated by ART against the reference genome of human Hg19, the differences between simulated reads and the reference genome are mismatches rather than gaps. With low penalty for gapped alignment, a gapped alignment may gain a high mapping quality score, which will damage the accuracy of the alignment.

**Table 3:** The number of TP (true positive), and FP (false positive) in the re-aligned reads from single-end simulated datasets.

	50-bp			75-bp			100-bp		
			
	#*RA*	#*TP*	#*FP*	#*RA*	#*TP*	#*FP*	#*RA*	#*TP*	#*FP*
RAUR(BWA)	312,949	284,796	28,153	658,289	629,733	28,556	2,222,453	2,197,995	24,458
RAUR(Bowtie2)	632,552	519,586	112,966	388,654	293,101	95,553	593,629	532,641	60,988

#RA is the number of confidently re-aligned reads with mapping quality not less than 10, #TP is the number of confidently and correctly re-aligned reads, and #FP is the number of confidently but incorrectly re-aligned reads.

**Table 4:** The number of TP (true positive), and FP (false positive) in the re-aligned reads from paired-end simulated datasets.

	50-bp			75-bp			100-bp		
			
	#*RA*	#*TP*	#*FP*	#*RA*	#*TP*	#*FP*	#*RA*	#*TP*	#*FP*
RAUR(BWA)	57,206	53,440	3,766	79,892	77,913	1,979	719,859	709,445	10,414
RAUR(Bowtie2)	123,759	108,171	15,588	138,273	126,500	11,773	223,491	209,981	13,510

#RA is the number of confidently re-aligned reads with mapping quality not less than 10, #TP is the number of confidently and correctly re-aligned reads, and #FP is the number of confidently but incorrectly re-aligned reads.

Taken RAUR(bowtie2) for example, the influence of different parameter *K *on the *Alignment rate *and *Precision *is analyzed in Table 5. We run RAUR(Bowtie2) with different initial values of parameter K, and the *Alignment rate *and *Precision *are compared, as shown in Table S3. The *Alignment rate *of RAUR(Bowtie2) are increased with larger initial values of *K*, while the *Precision *of RAUR(Bowtie2) are decreased. In the original mappings of Bowtie2, there are reads unmapped or mapped with low mapping qualities due to the exceeding of allowable mismatches or gaps. RAUR employs a parameter *K *to control the possible mismatches, therefore the *Alignment rate *are improved by RAUR(Bowtie2). For large initial values of K, the gapped alignments of Bowtie2 may damage the *Precision *of RAUR(Bowtie2). For smaller initial values of *K*, the *Precision *of RAUR(Bowtie2) are higher, because the lengths of reads trimmed with the small initial value of *K *are short, and most part of the trimmed reads are aligned to the genome in an ungapped fashion using the FM Index by Bowtie 2. However, for real data, the *Precision *of RAUR(Bowtie2) will be higher compared with those on simulated data. Because besides the substitution errors introduced by sequencers, the indels and substitutions will be introduced by the differences between the donor and reference genomes, gapped alignments performed by Bowtie2 will be useful. The influence of different initial values of *K *on the performance of RAUR(BWA) will be similar, which the *Alignment rate *of RAUR(BWA) are increased with larger initial values of *K*, but the *Precision *of RAUR(BWA) will not be decreased as much as RAUR(Bowtie2). The reason lies in the different strategies of Bowtie2 and BWA to perform gapped alignment, and BWA pays different penalties for mismatches, gap opens and gap extensions.

**Table 5:** The alignment rate and precision of Bowtie2 on single-end simulated data with different initial values of *K*.

	50-bp		75-bp		100-bp	
			
*K*	*Align.Rate*(%)	*Prec*(%)	*Align.Rate*(%)	*Prec*(%)	*Align.Rate*(%)	*Prec*(%)
10	0.870362	0.981524	0.858727	0.981852	0.834774	0.981529
9	0.869464	0.981703	0.856311	0.982638	0.832754	0.982217
8	0.868262	0.982009	0.853695	0.983442	0.830495	0.982984
7	0.866564	0.982608	0.85074	0.984281	0.827873	0.983787
6	0.864261	0.983586	0.847368	0.985199	0.824662	0.984629
5	0.861039	0.985146	0.84329	0.98618	0.820193	0.985421
4	0.856596	0.987344	0.837615	0.987297	0.812949	0.986246
3	0.850384	0.990075	0.828366	0.988768	0.800774	0.987542
2	0.841539	0.993	0.813271	0.991168	0.782962	0.990233
1	0.825283	0.995516	0.794526	0.994829	0.763764	0.993865

There are 7,740,912 simulated single-end reads with length 50-bp, 5,156,962 with length 75-bp, and 3,868,843 with length 100-bp.

### Real data and performance

To assess the performance on real data, each alignment program is run on three datasets of single-end reads (ERR008838(76-bp), SRR006273(76-bp) and ERR008843(83-bp)) and three datasets of paired-end reads (ERR007641(51-bp), SRR019044(76-bp), and ERR050728(90-bp)). The single-end reads were produced by Illumina for NA18633, NA18498, and NA18624 individuals, and the paired-end reads were produced by Illumina for NA12282, NA11831, and HG00759 individuals, included in the 1000 Genomes Project http://www.1000genomes.org. These reads are mapped to the human genome UCSC Hg19. The comparison of *Alignment rate *of different alignment programs on different datasets are shown in Table 6 and 7.

**Table 6:** The *alignment rate *and *precision *of each alignment method on single-end real data with different read length.

	*Alignment Rate*		
	
	SRR006273(76 bp)	ERR008838(76 bp)	ERR008843(83 bp)
BWA	69.0456	77.1065	81.1342
BWA(mem)	75.3120	80.3020	83.2176
RAUR(BWA)	83.0732	83.8950	86.3092
Bowtie2	70.6717	78.7660	82.3776
Bowtie2(local)	80.6902	85.9375	88.2963
RAUR(Bowtie2)	81.0039	83.8124	86.0461

There are 10,685,743 single-end reads with length 76-bp in SRR006273, 12,564,212 with length 76-bp in ERR008838, and 15,929,373 with length 83-bp in ERR008843.

**Table 7:** The *alignment rate *and *precision *of each alignment method on paired-end real data with different read length.

	*Alignment Rate*		
	
	ERR007641(51 bp)	SRR019044(76 bp)	ERR050728(90 bp)
BWA	82.7438	80.9078	95.7637
BWA(mem)	-	82.3725	96.1319
RAUR(BWA)	84.9410	85.8255	96.0608
Bowtie2	80.1309	79.8907	94.0414
Bowtie2(local)	-	87.4895	96.3645
RAUR(Bowtie2)	82.7563	86.2557	94.4253

There are 2,977,726 pairs reads with length 51-bp in ERR007641, 9,661,679 pairs with length 76-bp in SRR019044, and 676,633 pairs with length 90-bp in ERR050728.

In Table 6 the *Alignment rate *of RAUR(BWA) and RAUR(Bowtie2) are significantly higher than those of BWA and Bowtie2, and consistent with those of RAUR(BWA) and RAUR(Bowtie2) on single-end simulated data with read length 75-bp. A little different from the simulated results, the *Alignment rate *of RAU-R(BWA) and RAUR(Bowtie2) outperform those of BWA(men) on three datasets, while Bowtie2(local) gains the highest *Alignment rate *on SRR006273 and ER-R00884s3, compared with other alignment programs, which is 2% more than those of RAUR(BWA) and RAUR(Bowtie2).

On the three real datasets of paired-end reads, as shown in Table 7 RAUR(BWA) and RAUR(Bowtie2) outperform BWA and Bowtie2, and show significant improvement on ERR007641 and SRR019044. All the alignment programs work well on long reads (ERR050728(90-bp)). The *Alignment rate *of RAUR(BWA) is comparative with those of BWA(men) and Bowtie2(local), while the *Alignment rate *of RAUR(Bowtie2) is about 1-2% less than Bowtie2(local).

## Discussion

For a read, if it originates from a unique region and its differences with the reference sequence do not exceed the alignment tools' allowance, it will be mapped uniquely. If a read is copied from a repeat region within the allowed number of mismatches, it has multi hits and the alignment tools have little confidence in its mapping. However, a read is probably unmapped if it has too much mismatches in the alignment, no matter they are sequencing errors or variances. RAUR is proposed to re-align these reads which cannot be mapped by alignment tools. The trimming strategy adopted in RAUR is used to find out the longest and confident fragments of these unmapped reads, with *K *low quality bases at most. Therefore, compared with the original reads, the possible mismatches in the alignments of the trimmed reads will decrease, and the possibility of successful alignments will increase.

RAUR is not only efficient to re-align the unmapped reads, but also works well on the reads with low mapping quality scores. There exists some reads with multi hits, but in fact they come from the unique regions of the genome. Even for the repeat regions, two repeats of one type also have small differences. To uniquely map the reads in the repeat regions is also possible, if the characterized differences are involved in the alignment, rather than the sequencing errors. Our method can control the possible mismatches and emphasize the characterized differences in the alignment. Thus, for these reads with low mapping quality scores, RAUR can figure out their longest and confident fragments and try to find out their correct positions.

RAUR also can efficiently align long reads against a reference sequence, which is a new challenge to many alignment tools. As we known, the length of reads coming from the new sequencing technologies become longer and longer[24], which makes many of the alignment tools exclusively designed for reads no longer than 100 bp inefficient. However, RAUR can employ these short read alignment tools to align long reads.

## Conclusion

In this paper, by analyzing the base quality distributions of sequencing errors, a method (RAUR) is proposed to re-align the unmapped reads and the reads with low mapping quality scores. The key strategy adopted in our method is to align the most reliable and informative part of the read. We evaluate the method by comparing the *Alignment rate*s and *Precision *on both simulated data and real data with different lengths. Combined with BWA or Bowtie2, RAUR can align more reads confidently than BWA and Bowtie2, with comparative *Precision*. Furthermore, the performance of RAUR is seldom affected with the increasing of read length. Moreover, RAUR outperforms BWA-MEM and the local mode of Bowtie2 in some cases.

## Competing interests

The authors declare that they have no competing interests.

## Authors' contributions

XP and JW design the algorithm and implement it. XP and ZZ test it on different datasets. XP and QX evaluated the results. JW and XP drafted the manuscript together. ML and YP participated in revising the draft. All authors have read and approved the manuscript.

## Declarations

The publication costs for this article were funded in part by the National Natural Science Foundation of China under grant nos. 61232001, 61379108, and 61370172, Hunan Provincial Innovation Foundation For Postgraduate (CX2013B070), and Science and Technology Plan Projects of Science and Technology Bureau of Hengyang City (grant 2013KJ29).

This article has been published as part of *BMC Bioinformatics *Volume 16 Supplement 5, 2015: Selected articles from the 10th International Symposium on Bioinformatics Research and Applications (ISBRA-14): Bioinformatics. The full contents of the supplement are available online at http://www.biomedcentral.com/bmcbioinformatics/supplements/16/S5.
